# The Effects of Fortification of Legumes and Extrusion on the Protein Digestibility of Wheat Based Snack

**DOI:** 10.3390/foods5020026

**Published:** 2016-04-06

**Authors:** Swapnil S. Patil, Margaret A. Brennan, Susan L. Mason, Charles S. Brennan

**Affiliations:** Centre for food Research and innovation, Department of Wine, Food and Molecular Biosciences, Lincoln University, Canterbury 7647, New Zealand; SwapnilShamrao.Patil@lincolnuni.ac.nz (S.S.P.); Margaret.Brennan@Lincoln.ac.nz (M.A.B.); Sue.mason@lincoln.ac.nz (S.L.M.)

**Keywords:** protein digestibility, legumes, wheat, extrusion

## Abstract

Cereal food products are an important part of the human diet with wheat being the most commonly consumed cereal in many parts of the world. Extruded snack products are increasing in consumer interest due to their texture and ease of use. However, wheat based foods are rich in starch and are associated with high glycaemic impact products. Although legume materials are generally rich in fibre and protein and may be of high nutritive value, there is a paucity of research regarding their use in extruded snack food products. The aim of this study was to prepare wheat-based extrudates using four different legume flours: lentil, chickpea, green pea, and yellow pea flour. The effects of adding legumes to wheat-based snacks at different levels (0%, 5%, 10%, and 15%) during extrusion were investigated in terms of protein digestibility. It was observed that fortification of snacks with legumes caused a slight increase in the protein content by 1%–1.5% w/w, and the extrusion technique increased the protein digestibility by 37%–62% w/v. The product developed by extrusion was found to be low in fat and moisture content.

## 1. Introduction

Snack products are becoming an important part of the human diet as their convenience and availability attract consumer attention [1,2,3]. Most of the available snacks are made from refined cereal flours that are rich in salts, saturated fats and easily digested carbohydrates [3,4]. Cereals are the main source of carbohydrates in our diet. Barley, wheat, rice and maize are now gaining importance as they are rich sources of protein, dietary fibers and lipids. Cereals are the main source of energy (56%) for humans in some parts of the world [5]. It may be argued that the increase in the consumption of snack food products has led to an increase in obesity and thus an unhealthy population [3,4,6,7].

In terms of snack food products, it could be argued that legume grains and flours are underutilised in the extrusion process [8,9]. Legumes such as chickpea, lentils and soybean are an important source of protein for human body, particularly in parts of the world where meat and milk consumption is constrained by factors such as low availability, ethical reasons or allergenicity. Some researchers suggest that legume based products are essential in our daily diet for leading a healthy life [10,11]. From a nutritional point of view, legumes are of special interest because they are rich in dietary fibres [9] and protein [10]. Albumin and globulins are the dominant protein found in legume seeds, around 70% of legume protein is produced by globulins [12,13,14]. Legumes also contain considerable amount of vitamins and other micronutrients. The composition of legume materials are known to play a key role in preventing metabolic diseases such as diabetes mellitus [11,14,15] and coronary heart diseases [15,16]. It may therefore be possible by blending wheat grains and legume grain material to manufacture extruded snack products that have lower starch contents but higher protein contents, and potentially greater protein digestibility.

Thus, using starch sources such as maize, oat, barley and wheat combined with sources of protein such as peas or beans, can increase the nutritional quality of snack products. A combination of cereals and legumes can produce nutrition rich products [16]. Tiwari *et al*. [17] studied the addition of pigeon pea to wheat flour based biscuits and, Hara *et al*. [18] studied the effect of addition of legume flour to traditional cereal based flours and both research teams illustrated that an increase in protein content and potential nutritional improvements including an increase in protein digestibility. Another study by Madhumita and Prabhasankar [19] improved the nutritional value of pasta by adding black gram flour and also reported that the processing of food material increases value and shelf life of product.

There are many processing techniques such as milling, cooking, soaking, fermentation and extrusion, which, when implemented, help improve the nutritive value of food products [20]. Extrusion technique is one of the most common and popular processing techniques among the manufacturers due to its convenience and affordability and its importance has been widely accepted by the scientific community [3,20,21]. A broad range of snack foods and breakfast cereals can be generated using extrusion. High temperature, short time and high pressure are the common conditions for extrusion [3,20] such that extrusion cooking changes the biochemical properties of food. Extrusion can be used to produce innovative products such as cereal-based snacks, precooked breakfast cereals, modified starch and beverages [22].

Recently, there has been a growing emphasis on increasing the nutritional value of product creating a need for research on aspects such as understanding the *in vitro* digestibility of combined blends. A variety of protein and cereal sources have been utilised in order to improve the nutritional quality of extruded snack products [23,24,25,26,27,28]. The main aim of this study is to investigate whether the processing of food materials would affect the *in vitro* protein digestibility.

## 2. Experimental Section

### 2.1. Materials

Wheat grain was obtained from Champion flour mills (Christchurch, New Zealand), while lentil, yellow pea, green pea and chickpea pulses were obtained from supermarket local supplier (Foodstuffs NZ, Lincoln, New Zealand). Pepsin (1031 U/mg) from porcine gastric mucosa, albumin bovine serum, minimum 98% electrophoresis, was purchased from Sigma Aldrich (St. Louis, MO, USA) pancreatin (350 U/g) from porcine pancreas, was purchased from AppliChem Chemica Synthesis, Germany. Biorad Kit (Cat. #500-0006) for the Bradford assay was obtained from Bio-Rad Laboratories Inc. United States. All other chemical used were of analytical grade.

### 2.2. Methodology

#### 2.2.1. Extrusion

Different legumes were used as whole grains at 0%, 5%, 10%, and 15% replacement levels for wheat grain in the production of extrudates. Extrusion was conducted in a single screw extruder through a 3 mm die face and collected as collets (Millbank Ltd, Auckland, New Zealand). The extrusion parameters of screw speed (210 ± 5 rpm), temperature in the barrel (180 °C) and moisture content of samples (12%) were kept constant for all samples during the extrusion process and are illustrated in Table 1. Extruded products were collected and stored in airtight containers for further analysis.

#### 2.2.2. Moisture

The standard moisture determination method given by Approved Methods of the American Association of Cereal Chemists (1995) was used with a slight modification to measure the moisture content of the sample. Samples were dried in an oven at 105 °C overnight.

Moisture% = (W2 − W3)/(W2 − W1) × 100

where W1 = Weight of empty crucible, W2 = weight of crucible and sample before drying, and W3 = Weight of crucible and sample after dry.

#### 2.2.3. Protein Determination

Seeds were ground to fine powder by using grinder (Breville BCG 200, Breville, Auckland, New Zealand) the flour was then used to measure protein digestion for the raw samples. Protein content of the extrudates and raw flour samples before digestion was determined using the Dumas method (element analyser Model Vario MAX CN, Hanau, Germany). Protein estimation of digested sample was carried out by the Bradford method as mentioned previously [29].

#### 2.2.4. Fat

Crude fat was determined using BUCHI Soxhlet Extraction Unit E-816HE [30].

#### 2.2.5. *In Vitro* Protein Digestibility

*In vitro* protein digestibility was mainly adopted from Chen, 2002 [31] with slight modification. Sample weight was 2% w/v, measured and diluted in RO water. The pepsin (4 units/mg protein basis) was added after adjusting the pH to 2.0 with 1 M HCl. The solution was incubated at 37 °C for 60 min. After incubation, the pH was adjusted to 7.0 using 1 M NaOH. Pancreatin (4 units/mg protein basis) was added and digestion volume made to 50 mL. Samples were then incubated at 37 °C for 120 min. Aliquots were taken at 0, 60, 120 and 180 min intervals and placed in ice to stop enzyme activity, and then centrifuged at 3000 rpm for 5 min. The supernatant was collected for analysis. This method has several advantages over other methods, such as less time consuming, can also be applied to various protein samples and sufficiently sensitive to detect the effect of processing. After digestion, the remaining protein was determined by using Bradford method with slight modification [22,29]. The per cent digestibility was calculated as the difference between protein content at 0 min and after 180 min as a percentage of original protein content.

#### 2.2.6. Statistical Analysis

All sampling was performed in triplicate. The data were analysed by using ANOVA using Minitab software (version 16). Tukey’s test was applied to establish the level of significance (*p* < 0.05).

## 3. Result and Discussion

Legumes are regarded as grains that contain high protein and high fibre [8,13,17]. The results shown in Table 2 indicate that the protein content of legumes was considerably higher than that of wheat. It ranged from 20.27% to 25.33% for legumes, whereas that for wheat was 14.47%. Our results confirm the previous studies on the use of legume protein for industrial purposes [16,17].

### 3.1. Proximate Analysis of Extrudates

The moisture content of the extrudates (Table 3) showed slight variation, the highest value observed was wheat + 10% yellow pea (9.36%), whereas wheat + 15% lentil (7.56%). Previous research has illustrated that variations observed in moisture content of extruded products may be dependent on the feed moisture and the extrusion temperature [3,9,17]. Research has also suggested that raw material high in fibre content (such as brans or legumes) also contributes to an increase in water holding capacity and hence moisture content of the final product [3,17]. When moisture is retained by the extrusion process this can have a serious effect in consumer acceptability of cereal foods containing high fibre ingredients, for instance bulk density and overall product hardness [4]. In this study the presence of legumes in the extruded formulations had no effect on product moisture of the extruded collets (Table 3).

The fat content in all samples were less than 1% except wheat + 10% green pea (1.03%). The lowest fat content was shown by wheat + 15% lentil. There was no significant difference between the samples in the concentration of fat, indicating that it is possible to include legumes into cereal extrudates without affecting the nutritional fat content of the foods (Table 3).

However, protein values (Table 3) show that the addition of legume grains to the extruded samples increases the protein content of all the products. All combined samples showed significantly (*p* < 0.05) higher protein content than the control samples. Wheat + 15% yellow pea and wheat + 15% green pea showed significantly higher protein content than control sample and other combinations. The obtained results indicate that adding different amount of legumes to wheat based extrudates have significantly increased the protein content. Similar results were obtained by Gularte *et al*. [32], with the addition of 50% of different legumes (Chickpea, lentils, bean and pea) to the rice based gluten free layer cakes increasing the protein content. Pastor-Cavada *et al*. [33] also observed increase in corn and rice based extrudate samples after adding legumes. Similarly, Zucco [34] reported that adding wild legumes to wheat based cookies increases the protein levels of the cookies.

### 3.2. In Vitro Protein Digestibility

*In vitro* protein digestibility (IVPD) of raw flour mix and extrudates are given in Table 4. The method used in this research was adapted from that previously used Chen *et al*., 2002 [31]. Although the protocol was not supported by *in vivo* determinations in our experiment (due to facility constraints) Chen *et al*., in their manuscript provide a review of the method in relation to *in vivo* analysis of protein digestibility. The results show that IVPD of raw flour mix was less than that of extrudates. The IVPD of the control samples was relatively low as compared to other combinations in both raw flour mixes and the extrudates (31.60% and 59.26%, respectively). In samples containing only the raw flour mixes, the highest level of digestibility was observed in wheat + 5% green pea. However, in the extruded samples, the wheat + 15% green pea product showed the highest level of protein digestibility. No clear significant differences were observed in samples with increased legume content, for instance extruded wheat samples with 5% lentil addition appeared to be not significantly different in their protein digestibility level than those samples with 15% lentil addition. The values clearly indicate the effect that extrusion processing has on the protein digestibility of the samples with significant increases in protein digestibility being observed after extrusion processing. For instance, the extrusion process itself generally doubled the digestibility of protein within the samples. This is in line with previous results obtained in terms of carbohydrate digestibility, which have illustrated that the effect of both shear and expansion on starch results in a higher glycaemic index food material [3,4]. Previous research has indicated that the presence of antinutritional compounds such as tannin can decrease the protein digestibility [35], however this was not observed in our results, possibly due to the fact that the mechanical and chemical processing factors from extrusion technology play a larger role in protein digestibility than the limiting behaviour of anti-nutritional factors. There may also be some other factors such as grain structure and cell wall components of the seed that can affect the solubility and digestibility of protein in seed, also protein could reacts with non-protein components present in seed during processing and it possibly leads to digestibility rates [36]. For instance, Linsberger *et al*. [37] reported that applying pressure and cooking to legume seeds at a high temperature increases the protein digestibility of such legume seeds, possibly by increasing the solubility of the protein and fragmenting the long polymer chins of intact proteins. Abd El_Hady and Habiba [38] observed an increase in the protein digestibility of legumes by extrusion technique. The rise could be related to the degradation of the protein complexes within the extruded samples and the denaturation of protein due to the heat and shear. The alterations in protein structure thus make the extruded products more susceptible to degradation and hence the release of the products of digestion are increased—the bioavailability of the protein may be elevated. As mentioned previously, this is similar to the mechanism by which extrusion processing shears and denatures carbohydrate fractions leading to increased carbohydrate digestibility of extrudates [3,6,29]. It is therefore sensible to suggest that in this set of experiments the extrusion processing parameters have led to a denaturation of protein structures leading to increased ease of digestion.

## 4. Conclusions

It is concluded that adding legumes to wheat based snacks increases the nutritional quality of product. The research established that adding legumes increases the protein content of the product. The increasing demand of nutrition rich food by the rapidly growing population has increased the pressure on food processing and agricultural sector to produce food alternatives that provides nutrition and functional benefits to consumers and producers at an affordable price. This study also confirms that extrusion process increases the protein digestibility. In addition, the results showed that a product prepared using extrusion and adding legumes is low in fat. Considering the protein content and its digestibility, use of legumes has great potential for producing extrudate products of commercial value.

## Figures and Tables

**Table 1:** Extrusion parameter for the samples.

Sample	Torque	Shaft Speed (RPM)
Wheat	64	210
Wheat 5% Yellow pea	57	200
Wheat 5% Green pea	72	210
Wheat 5% Lentil	48	210
Wheat 5% Chickpea	46	210
Wheat 10% Yellow pea	53	200
Wheat 10% Green pea	70	210
Wheat 10% Lentil	48	210
Wheat 10% Chickpea	48	210
Wheat 15% Yellow pea	62	200
Wheat 15% Green pea	61	210
Wheat 15% Lentil	46	210
Wheat 15% Chickpea	44	210

**Table 2:** Protein content of raw ingredients used.

Sample	Protein *(g/100 g Dry Matter Basis)*
Wheat	14.47 ± 0.11
Lentil	25.33 ± 0.17
Chickpea	22.96 ± 0.24
Yellow pea	21.73 ± 0.13
Green pea	20.47 ± 0.28

**Table 3:** Proximate compositions of the wheat based extrudates (measured as g/100 g dry matter basis).

Sample	Protein	Fat	Moisture
Wheat	13.54 ± 0.04 ^e^	0.58 ± 0.09 ^c,d,e^	9.29 ± 1.89 ^a^
Wheat 5% Yellow pea	14.30 ± 0.11 ^b,c^	0.51 ± 0.06 ^e^	8.11 ± 0.21 ^a^
Wheat 5% Green pea	14.10 ± 0.04 ^c,d^	0.54 ± 0.01 ^e^	8.41 ± 0.64 ^a^
Wheat 5% Lentil	14.53 ± 0.21 ^b,c^	0.55 ± 0.01 ^e^	8.51 ± 0.26 ^a^
Wheat 5% Chickpea	14.25 ± 0.08 ^b,c^	0.62 ± 0.04 ^d^	8.13 ± 0.40 ^a^
Wheat 10% Yellow pea	14.96 ± 0.04 ^a^	0.72 ± 0.02 ^b,c,d^	9.36 ± 0.76 ^a^
Wheat 10% Green pea	14.57 ± 0.05 ^b,c^	1.03 ± 0.11 ^a^	9.05 ± 0.90 ^a^
Wheat 10% Lentil	14.59 ± 0.23 ^b,c^	0.83 ± 0.09 ^b^	8.62 ± 0.41 ^a^
Wheat 10% Chickpea	14.28 ± 0.06 ^c^	0.90 ± 0.10 ^a,b^	7.64 ± 0.35 ^a^
Wheat 15% Yellow pea	15.16 ± 0.17 ^a^	0.75 ± 0.04 ^b,c^	7.75 ± 0.13 ^a^
Wheat 15% Green pea	14.79 ± 0.02 ^b^	0.75 ± 0.08 ^b,c^	8.42 ± 0.76 ^a^
Wheat 15% Lentil	15.05 ± 0.03 ^a^	0.72 ± 0.05 ^b,c,d^	7.56 ± 0.14 ^a^
Wheat 15% Chickpea	14.47 ± 0.11 ^b,c^	0.29 ± 0.01 ^f^	7.75 ± 1.53 ^a^

* Values with different letters are significantly different in a same column (*p* < 0.05)

**Table 4:** Protein digestibility of raw flour mix and extrudates (given as a % of total protein in samples as determined by the Dumas method).

Sample	Raw Mix	Extrudates
Wheat	31.60 ± 2.66 ^b,c^	59.26 ± 1.08 ^d^
Wheat 5% Yellow pea	32.70 ± 2.04 ^b,c^	63.39 ± 0.73 ^a,b,c^
Wheat 5% Green pea	38.23 ± 2.11 ^a^	62.95 ± 0.72 ^b,c^
Wheat 5% Lentil	29.33 ± 0.48 ^c^	63.27 ± 0.20 ^b,c^
Wheat 5% Chickpea	29.97 ± 1.11 ^b,c^	61.44 ± 0.43 ^b,c^
Wheat 10% Yellow pea	29.27 ± 2.86 ^b,c^	65.50 ± 1.49 ^a,b^
Wheat 10% Green pea	28.92 ± 1.17 ^b,c^	64.03 ± 1.09 ^a,b^
Wheat 10% Lentil	32.00 ± 1.49 ^b,c^	62.46 ± 1.13 ^b,c^
Wheat 10% Chickpea	31.30 ± 0.64 ^b,c^	60.69 ± 1.29 ^c,d^
Wheat 15% Yellow pea	31.59 ± 3.38 ^b,c^	65.61 ± 1.45 ^a^
Wheat 15% Green pea	33.02 ± 2.18 ^b,c^	65.69 ± 0.32 ^a^
Wheat 15% Lentil	31.85 ± 1.55 ^b,c^	62.26 ± 0.74 ^b,c^
Wheat 15% Chickpea	35.21 ± 0.92 ^b,c^	62.46 ± 0.97 ^b,c^

* Values with different letters are significantly different in a same column (*p* < 0.05).
